# Establishing the stability number descriptor for Fe–N–C fuel cell electrocatalysts†

**DOI:** 10.1039/d5sc00547g

**Published:** 2025-04-14

**Authors:** Yu-Ping Ku, Kavita Kumar, Antoine Bonnefont, Li Jiao, Marco Mazzucato, Christian Durante, Frédéric Jaouen, Serhiy Cherevko

**Affiliations:** a Forschungszentrum Jülich GmbH, Helmholtz-Institute Erlangen-Nürnberg for Renewable Energy (IET-2) Cauerstraße 1 91058 Erlangen Germany yupingku427@gmail.com s.cherevko@fz-juelich.de; b Department of Chemical and Biological Engineering, Friedrich-Alexander University Erlangen-Nürnberg Egerlandstraße 3 91058 Erlangen Germany; c Univ. Grenoble Alpes, Univ. Savoie-Mont-Blanc, CNRS Grenoble-INP, LEPMI 38000 Grenoble France; d Institut Charles Gerhardt Montpellier, Univ. Montpellier, CNRS, ENSCM 1919 route de Mende F-34293 Montpellier France; e Department of Chemical Sciences, University of Padova via Marzolo 1 35131 Padova Italy

## Abstract

Fe–N–C electrocatalysts demonstrate high potential in catalyzing oxygen reduction reaction (ORR) in polymer electrolyte fuel cells, yet the bottleneck for their application is their moderate stabilities. In our previous work, we discovered a linear correlation between the rates of ORR and Fe dissolution in alkaline media at room temperature, and the stability (*S*-) number descriptor that reflects this correlation was introduced. On the way toward further generalization and establishment of this descriptor, we investigate the effect of pH, potential, current density, and temperature on the dissolution behavior of various representative Fe–N–C electrocatalysts. It is shown that the *S*-number concept is also applicable for ORR and Fe dissolution in alkaline electrolytes at 70 °C. It is more challenging to apply the *S*-number in acidic media, where the *S*-number is a function of ORR current density. A kinetic model is introduced, showing that the local pH inside the catalyst layer rises significantly with increasing current densities. The pH dependence of the *S*-number explains the results in acidic electrolytes. Accounting for such a dependence, the *S*-number descriptor can also benchmark Fe–N–C stability in acidic electrolytes. It is considered that this concept can also be extended to other reactions, allowing more rational activity and stability screening of electrocatalysts.

## Abstract 2

For Fe–N–C catalysts, the stability (*S*-) number hardly varies in basic media. While in acidic media, the *S*-number stays constant at low current densities, and then increases with the climbing current density, likely due to the rising local pH values.

## Graphical abstract

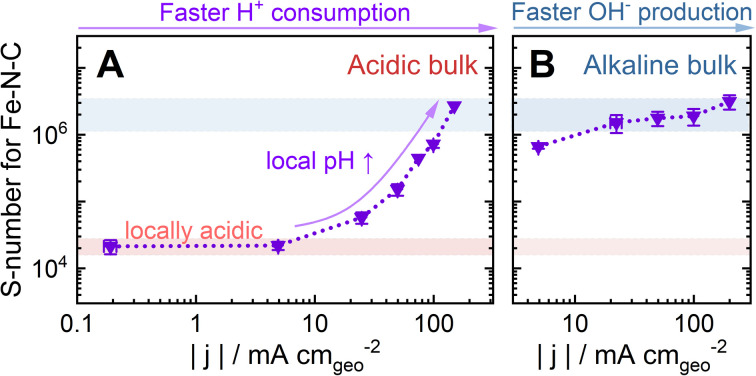

## 1. Introduction

Fe–N–C catalysts are the most promising alternative to platinum-group-metal (PGM) materials used as the oxygen reduction reaction (ORR) catalysts at cathodes of proton exchange membrane fuel cells (PEMFCs) and anion exchange membrane fuel cells (AEMFCs). However, Fe–N–C materials still suffer from unsatisfactory durability, particularly in acidic environments of PEMFCs. In order to develop mitigation strategies that improve their durability, the fundamental understanding of the degradation mechanisms still needs to be deepened. Hence, the current knowledge of the degradation mechanisms of Fe–N–C catalysts has been summarized in detail in ref. 1 and briefly presented here as follows.

The relevant potential (*E*) ranges for ORR catalysts are 0.6 V_RHE_ < *E* < open circuit potential (OCP) during the operating mode and OCP < *E* < 1.5 V_RHE_ in start–stop events.^2^ The primary degradation mechanism of Fe–N–C catalysts in the *E* range OCP < *E* < 1.5 V_RHE_ is the electrochemical oxidation of the N–C matrix,^3–8^ producing either O-containing functional groups or gaseous NO_*x*_/CO_*x*_. The compromised integrity of the N–C matrix may lead to Fe demetallation,^8,9^ followed by partial re-deposition as iron oxides,^8^ and the destruction of the catalyst layer's porous structure.^10^ On the other hand, the main degradation mechanisms during ORR (0.6 V_RHE_ < *E* < OCP) are the reactive oxygen species (ROS) attack^11–13^ and Fe demetallation (or Fe leaching/dissolution).^9,14–16^ The ROS are usually produced by Fenton reactions between Fe species and the ORR intermediate/byproduct H_2_O_2_. The ROS could oxidize the carbon surface (faster in acidic than in alkaline media, due to different ROS species formed at different pH), resulting in a decreased turnover frequency (TOF) of FeN_*x*_C_*y*_ sites.^11–13^ Additionally, the higher TOF contributed by the more basic N-groups after NH_3_-pyrolysis may reduce due to their protonation and subsequent anion adsorption.^17^ Besides a decreased average TOF of FeN_*x*_C_*y*_ sites, the demetallation of Fe from such sites during operation can decrease the active site density (SD) and thus also decrease the ORR activity of the active layer. Fe demetallation can be indirectly triggered by the electrochemical oxidation of the N–C matrix (OCP < *E* < 1.5 V_RHE_) or directly induced by the ORR (0.6 V_RHE_ < *E* < OCP). The rate of Fe dissolution in the *E* range 0.6 V_RHE_ < *E* < OCP could vary with the chemical nature of the Fe species,^14,16,18^ the pH value of the environment,^14^ the electrochemical potential,^9,15^ the temperature,^9^ and the presence/absence of O_2_.^15^ A fraction of dissolved Fe species might re-deposit on the catalytic surface as Fe oxide clusters.^16,19,20^ Such clusters have some ORR activity in alkaline media, and may have synergy with FeN_*x*_C_*y*_ sites.^19^ Consequently, the dominant degradation mechanisms in the operating mode of PEMFCs are the ROS attack and Fe dissolution. In contrast, the main degradation mechanism in AEMFCs in operation is Fe dissolution, while deposition as Fe oxides could contribute to mitigating the ORR activity loss.^19^

Hence, in studies on the durability of Fe–N–C catalysts, the fate of the active Fe species has been one of the main focuses. The initial active Fe species can be diverse, including atomically dispersed Fe in N–C structure (FeN_*x*_C_*y*_ sites), Fe-carbide particles protected by N-doped graphene layers (Fe_3_C@N–C), *etc.* As these active Fe species possess varied activities and stabilities, Fe–N–C catalysts with mainly a certain type of Fe species are ideal for fundamental studies. Furthermore, the changes in the Fe species in a membrane electrode assembly can be observed post-mortem by Mössbauer spectroscopy. Alternatively, Fe dissolution can be detected *in situ* by coupling an electrochemical cell, such as a scanning flow cell (SFC) or a gas diffusion electrode (GDE) half-cell, to an inductively coupled plasma mass spectrometer (SFC-ICP-MS^9,14^ or GDE-ICP-MS,^8,15,21^ respectively), revealing the dissolution rate as a function of the applied electrochemical protocol.

In order to characterize the activity *vs.* stability of a given electrochemical catalyst, the stability (*S*-) number was first introduced for Ir-based catalysts for oxygen evolution reaction (OER) and defined as the ratio between the amount of evolved O_2_ and the amount of dissolved iridium.^22^ The higher the *S*-number is, the more O_2_ can be evolved per dissolved iridium. Moreover, the *S*-number was found to be relatively constant over wide ranges of current density and potential, suggesting that the Ir dissolution and OER mechanisms are interconnected, potentially through a common intermediate.^23^ In this line, a linear correlation between the rates of Fe dissolution and charge transfer during ORR at current densities up to −125 mA cm^−2^ was revealed for both Fe_3_C@N–C-rich and FeN_*x*_C_*y*_-rich Fe–N–C catalysts in alkaline media at room temperature (RT) in the potential range 0.57–0.87 V_RHE_, in our previous work using GDE-ICP-MS.^15^ Based on this observation, the *S*-number descriptor was also introduced for Fe–N–C catalysts in the testing condition and defined as the number of electrons exchanged in the ORR per dissolved Fe cation. In our previous work,^15^ we reported the ORR charge-normalized amount of dissolved Fe species, which is inversely proportional to the *S*-number.

The *S*-number can be a powerful stability descriptor for Fe–N–C catalysts, especially at the beginning of life when their states are still close to their pristine states, for which the Fe species can be well identified from *ex situ* characterization of the catalyst or active layer. When the dissolved Fe is mainly from the most active Fe species, such as the FeN_*x*_C_*y*_ sites, the *S*-number represents an average of how many times ORR faradaic charge transfer occurs among a large amount of FeN_*x*_C_*y*_ sites before an Fe ion dissolves from one of the active sites. However, after a long aging protocol or accelerated stress test (AST), the amount of dissolved Fe species can be significant, and the amount of re-deposited Fe species as well. In turn, the Fe dissolution from such inactive (or less active) redeposited Fe species could then contribute to the amounts of leached Fe measured after the aging protocol or AST. The resulting *S*-number measured on such a modified Fe–N–C surface might then no longer correspond to the stability of the FeN_*x*_C_*y*_ sites. Hence, interpreting *S*-numbers obtained from data measured before, during, and after ASTs must be proceeded with caution.

The observed constant *S*-number (*i.e.* the same linear correlation between the amount of the dissolved Fe and the number of electrons exchanged in the ORR) across a range of current densities for Fe–N–C materials in alkaline media suggests that the destabilization of the active Fe species is predominantly due to their less stable intermediate state(s) during the ORR catalytic cycle. It can be expected that the more dominant this demetallation mechanism is, the stronger the correlation between the amount of the dissolved Fe and the number of electrons exchanged in the ORR is (or the more constant the *S*-number is). In other words, if the *S*-number varies with the electrochemical potential or current density, then other degradation mechanisms that are potential-dependent or some current-density-dependent variables should be considered. For example, the faradaic efficiency of H_2_O_2_ could vary with potential,^11,24^ and so would the following ROS attack. The ROS may oxidize the matrix around FeN_*x*_C_*y*_ sites, which may not only reduce their TOF but also strengthen the Fe–N bonds of FeN_4_C_10_ sites or weaken the Fe–N bonds of FeN_4_C_12_ sites.^11,25^ Moreover, the oxidation state of the Fe of FeN_4_C_12_ sites switches from +III at 0.8 V to +II at 0.2 V, whereas that of FeN_4_C_10_ sites do not.^16^ Hence, the stability of FeN_4_C_12_ sites may be potential-dependent. As another example, an elevated ORR current density means faster consumption of H^+^ in acidic or production of OH^−^ in alkaline catalyst layers. If these processes are faster than the migration of H^+^ from, or OH^−^ to, the bulk electrolytes, then the local pH values in the catalyst layers would increase.^26^ The increased local pH can be a critical factor for the stability of FeN_*x*_C_*y*_ sites because they have been theoretically predicted^27^ and experimentally proven^14^ more stable in alkaline than in acidic media in the *E* range 0.6 < *E* < 1.0 V_RHE_. Hence, before using the *S*-number to compare Fe–N–C catalysts in other conditions, the linear correlation between the amount of dissolved Fe species and the number of electrons exchanged in the ORR in those conditions should be verified.

In this study, we investigate for the first time the use of the *S*-number for Fe–N–C materials in acidic and alkaline media, at both room and elevated temperatures, using GDE-ICP-MS. This stability descriptor was validated by correlating the amount of dissolved Fe with the number of electrons exchanged in the ORR. Relevant conditions, such as high current density (−100 mA cm^−2^), high temperature (70 ± 6 °C), and potential range 0.55–1.0 V_RHE_, were applied in the GDE setup. The study was systematically conducted on a commercial Fe–N–C catalyst from Pajarito Powder (made by silica hard template method), and on two laboratory Fe–N–C materials (one was derived from ZIF-8 and the other from steam-treated carbon black). While the materials differ by their synthesis methods, all three are rich in FeN_*x*_C_*y*_ sites and poor in Fe side-phases. Our results point toward the *S*-number as a suitable stability descriptor for Fe–N–C catalysts in alkaline media not only at RT but also at 70 °C. This descriptor could also be meaningful in acidic media if the impact of the increased local pH induced by a faster ORR rate is considered, as supported by experimental and kinetic modeling results in this study.

## 2. Experimental

### 2.1 Fe–N–C catalysts

There are three FeN_*x*_C_*y*_-rich Fe–N–C catalysts studied in this work: one commercial Fe–N–C catalyst (Pajarito Powder PMF-D14401, noted as PAJ_FeN_*x*_C_*y*_ in this work) and two laboratory-synthesized Fe–N–C catalysts, CNRS_FeN_*x*_C_*y*_ from CNRS – University of Montpellier (labeled as Fe_0.5_-dry in ref. 28), and UNIPD_FeN_*x*_C_*y*_ from University of Padova (labeled as FNCBSt10 in ref. 29). The synthesis of CNRS_FeN_*x*_C_*y*_ used a sacrificial metal–organic framework, ZIF-8, to obtain its porous structure.^28^ In contrast, the synthesis of UNIPD_FeN_*x*_C_*y*_ employed an activation procedure with steam on a commercial carbon black as the carbon support to adjust its porous structure.^29^ The ^57^Fe Mössbauer spectra of PAJ_FeN_*x*_C_*y*_ and CNRS_FeN_*x*_C_*y*_ were provided in ref. 8 and 28, respectively, testifying that the Fe species in these two catalysts are mostly FeN_*x*_C_*y*_ species. Moreover, a detailed characterization of UNIPD_FeN_*x*_C_*y*_ was provided in ref. 29, where its transmission electron microscopy images showed very few isolated Fe nanoparticles covered with carbon shells. Thus, UNIPD_FeN_*x*_C_*y*_ is also considered an FeN_*x*_C_*y*_-rich Fe–N–C catalyst.

### 2.2 Electrode preparation

In this work, when doctor-blade coating an Fe–N–C catalyst layer (CL), a certain thickness of a prepared ink was applied on a gas diffusion medium (H23C8, Freudenberg, 3 × 3 cm^2^), using an automated film applicator (ZAA 2300, Zehntner) where the plate was set to 30 °C. For the CLs tested in 0.1 M NaOH (Merck Suprapure), the ink was composed of 7.7 wt% of Fe–N–C catalyst (see Section 2.1), 3.3 wt% of a commercial ionomer (Aemion HNN5-00-X, Ionomr), and 89.0 wt% of 1-propanol (≥99.9%, Sigma-Aldrich). For the CLs tested in 0.1 M HClO_4_ (Suprapur, Sigma-Aldrich), the ink consisted of 6.2 wt% of Fe–N–C catalyst, 44.0 wt% of a commercial Nafion solution (D2021, Fuel Cell Store, containing 20 wt% Nafion), and 49.8 wt% of 2-propanol (Supelco, EMSURE). The ink preparation always started with thoroughly mixing the Aemion ionomer or Nafion solution with the (additional) solvent. Then, the Fe–N–C catalyst was added to this solution. The resulting ink was first stirred for one hour, then sonicated for one hour (100 W VWR Ultrasonic Cleaner USC 500 THD, temperature ≤30 °C), and finally stirred until being applied on the gas diffusion medium. The applied ink was then dried at 60 °C, under 1 atm in the first hour and under reduced pressure in the second hour. The catalyst loadings are provided in Table 1. The CLs with Aemion were kept in 1 M KOH (EMSURE^®^, Merck) for three hours to exchange the I^−^ in as-received Aemion with OH^−^, and the CLs with Nafion were kept in ultrapure H_2_O for one hour to wet the CLs, followed by a thorough washing step with ultrapure H_2_O repeated three times before the measurements.

**Table 1:** The testing conditions: the electrolyte and its temperature (*T*_electrolyte_); the gas purged from the side of the gas diffusion layer (O_2_, humidified or not), its temperature (*T*_gas_) and flow rate. The catalyst loading and the thickness of each catalyst layer (CL). The collection efficiency (CE) and the electrolyte flow rate during each measurement. The calibrated potential of the reference electrode (*E*_Ag/AgCl_) at *T*_electrolyte_. The details of the 200-cycle ASTs: two galvanostatic steps in each cycle, 3 seconds at “*j*” mA cm^−2^ and 3 seconds at −0.1 mA cm^−2^; and the potential *E* range resulting from the applied “*j*” mA cm^−2^

	UNIPD_FeN_*x*_C_*y*_		PAJ_FeN_*x*_C_*y*_				CNRS_FeN_*x*_C_*y*_			
Electrolyte	0.1 M NaOH		0.1 M NaOH		0.1 M HClO_4_		0.1 M NaOH		0.1 M HClO_4_	
*T*_electrolyte_, °C	Room temperature		67.0 ± 3.0		74.5 ± 0.5		71.0 ± 2.5		69.5 ± 2.5	
*T*_gas_, °C			67.0 ± 7.0		71.5 ± 1.5		71.5 ± 0.5		73.5 ± 1.5	
Humidification/O_2_ flow rate, mL min^−1^	Non-humidified/50		Humidified/125		Humidified/125		Humidified/125		Humidified/125	
Loading, mg_Fe–N–C_ cm^−2^	0.42	0.64	1.00	0.61	0.98	0.98	0.82	0.80	0.98	1.01
Thickness of CL, μm	10.8	15.0	37.0	24.8	42.3	43.5	28.0	25.0	30.5	29.8
CE, %	19.8	22.6	26.4	28.7	27.8	41.2	26.6	26.5	20.4	17.8
Electrolyte flow rate, mL min^−1^	0.18	0.18	0.23	0.22	0.24	0.24	0.23	0.21	0.22	0.21
*E*_Ag/AgCl_, V_RHE_	0.971 ± 0.001		0.944 ± 0.003		0.330 ± 0.001		0.943 ± 0.002		0.275 ± 0.011	
*j*, mA cm^−2^ in AST	−100		−100		−100		−100		−75	
*E*, V_RHE_ (resulting from the applied “*j*”)	0.50–0.60		0.60–0.64		0.68–0.74		0.59–0.63		0.50–0.62	

### 2.3 GDE-ICP-MS measurements

**Scheme 1:**
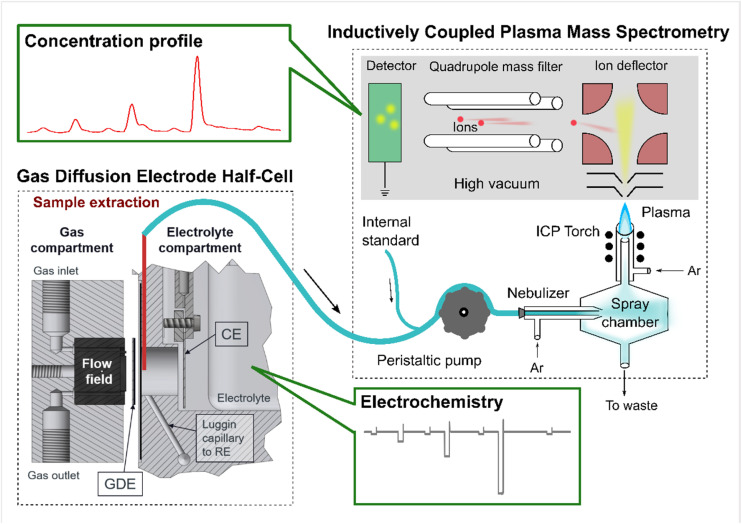
An illustration of the gas diffusion electrode (GDE) half-cell coupled to an inductively coupled plasma mass spectrometry (GDE-ICP-MS) setup. The scheme was originally adapted from Fig. S1 in ref. 30 (an open access article). This adapted scheme has been published in our previous work.^15^

To detect the online dissolution from a GDE sample during electrochemical protocols, the technique GDE-ICP-MS (ICP-MS: Perkin Elmer, NexION™ 350X) has been developed in our previous works,^8,15,30^ which provided the detailed methodology, including the calculation of the collection efficiency (CE) (see eqn (1)). The CE values in this work are listed in Table 1. A scheme of the GDE-ICP-MS setup is provided as Scheme 1, which was originally adapted from Fig. S1 in ref. 30 and was already published in ref. 15.

1 CE = *m*_ICP-MS_/(*m*_bulk,end_ − *m*_bulk,start_ + *m*_ICP-MS_ + *m*_tube_)

In eqn (1), *m*_ICP-MS_, *m*_bulk,end_ − *m*_bulk,start_, and *m*_tube_ refer to the amounts of the dissolved metal that were collected into the ICP-MS, accumulated in the bulk electrolyte, and stayed in the tube where the electrolyte recirculated during the measurement for a heating purpose, respectively.

The *S*-number is defined as the number of electrons exchanged in the ORR divided by the number of dissolved Fe, that is:

2 *S*-number = (|*j*| × *t* ÷ *e*)/(*n*_Fe,diss._ × *N*_A_)

In eqn (2), *j*, *t*, and *e* refer to current density, step duration, and elementary charge, respectively, and *n*_Fe,diss._ and *N*_A_ are the amount of the dissolved Fe in mole cm^−2^ and the Avogadro constant, respectively.

Moreover, for an ICP-MS using Ar plasma to detect ^56^Fe, operating the ICP-MS in dynamic reaction cell mode using CH_4_ (N45, Air Liquide) is required for a higher ratio of ^56^Fe to ^40^Ar^16^O^+^ (signal-to-noise ratio), as CH_4_ reacts with ^40^Ar^16^O^+^ faster than with ^56^Fe. The long-term status of the ICP-MS was tracked with an internal standard solution containing a constant concentration of ^74^Ge (100 μg L^−1^ for the measurements of UNIPD_FeN_*x*_C_*y*_ and 2.5 μg L^−1^ for the others, Merck Centripur) in 1 wt% HNO_3_ (ROTIPURAN^®^Supra, ROTH). Before every measurement, a four-point calibration curve was created with a blank electrolyte and three standard solutions (0, 1, 5, 25 μg_Fe_ L^−1^). The standard solutions were prepared in two steps. First, the Merck Centripur ICP standard solution (1000 mg_Fe_ L^−1^) was diluted to 1 mg_Fe_ L^−1^ with 1 wt% HNO_3_. Then, the 1 mg_Fe_ L^−1^ middle standard solution was diluted with the blank electrolyte to the desired concentrations.

**Scheme 2:**
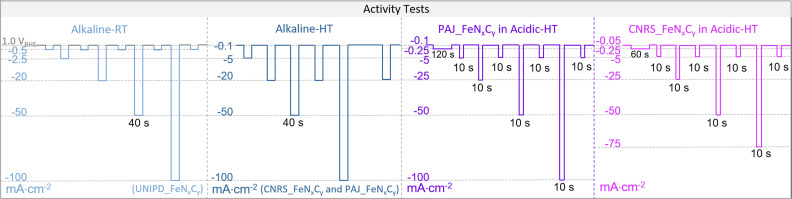
The electrochemical protocols of the activity tests from left to right are for UNIPD_FeN_*x*_C_*y*_ in alkaline media at room temperature (Alkaline-RT, light blue), for both CNRS_FeN_*x*_C_*y*_ and PAJ_FeN_*x*_C_*y*_ in alkaline media at 70 ± 6 °C (Alkaline-HT, dark blue), for PAJ_FeN_*x*_C_*y*_ in acidic media at HT (Acidic-HT) (violet), and for CNRS_FeN_*x*_C_*y*_ in Acidic-HT (magenta). Except for the potentiostatic steps at 1.0 V_RHE_ as the near OCP in the Alkaline-RT protocol (marked in grey), all others were galvanostatic steps with the applied current densities noted on the left side of each protocol. Apart from the near OCP steps (1.0 V_RHE_, −0.1 mA cm^−2^, or −0.05 mA cm^−2^) and the 120-s or 60-s steps at −0.25 mA cm^−2^ in Acidic-HT, the step durations of the others were set to 40 s, 40 s, and 10 s, in the activity test protocols in Alkaline-RT, Alkaline-HT, and Acidic-HT, respectively.

In this work, PAJ_FeN_*x*_C_*y*_ and CNRS_FeN_*x*_C_*y*_ were tested in both 0.1 M HClO_4_ and 0.1 M NaOH at 70 ± 6 °C (HT) (see Table 1) while UNIPD_FeN_*x*_C_*y*_ was tested in 0.1 M NaOH at RT. During each online measurement, O_2_ was provided from the gas-diffusion-layer side of the GDE sample with a flow rate, whose value is provided in Table 1. Meanwhile, an additional 50 mL min^−1^ Ar flow was purged into the electrolyte for stirring, which from experience is good for the signal-to-noise ratio of the online dissolution profiles. For all electrochemical protocols in the three testing conditions (Alkaline-RT, Alkaline-HT, and Acidic-HT), an activity test was always performed before and after a 200-cycle accelerated stress test (AST), each cycle of which consisted of two galvanostatic steps, 3 seconds at “*j*” mA cm^−2^ (the *j*-value for all experiments is listed in Table 1) and 3 seconds at −0.1 mA cm^−2^. For each condition, the activity test is illustrated in Scheme 2. The exact sets of chosen current densities in the activity tests vary slightly among the conditions to cover the potential and current density ranges that are relevant to fuel-cell applications. During the activity tests, the galvanostatic steps in Acidic-HT were shortened to only 10 s due to the recognized more rapid degradation of Fe–N–C catalysts in acidic media, while in Alkaline-HT and Alkaline-RT, the galvanostatic steps were prolonged to 40 s because of the lower signal-to-noise ratio in alkaline media. The measured electrochemical potential was 100% post-measurement *iR*-corrected, with respect to a reversible hydrogen electrode (RHE). For the *iR*-correction, the uncompensated resistance (*R*_u_) was obtained with electrochemical impedance spectroscopy at each applied current density. The calibration of the reference electrode Ag/AgCl (Metrohm, inner compartment: 3 M KCl, outer compartment: 3 M KCl for alkaline measurements or 0.1 M HClO_4_ for acidic measurements) with respect to a RHE was performed every day at the temperature of interest, of which the results are provided in Table 1. For the calibration of the reference electrode at an elevated temperature, the used electrochemical cell was put in a water bath that was maintained at the targeted temperature. The counter electrode was an expanded sheet of Ti coated with Ir/Ta mixed metal oxide (METAKEM). The geometric area of the GDE sample *S*_GDE_ was 2.01 cm^2^. To show the reproducibility of the result, each measurement was conducted twice, each time on a fresh GDE sample.

### 2.4 Kinetic modeling

The concentration profiles of proton in the aqueous phase (*C*_H_), Fe^III^ (*C*_Fe_) and hydroxides ions (*C*_OH_) as a function of time (*t*) and space (*x*) are modelled by solving a system of partial differential equations describing the effect of mass transport and of reactions in the catalyst layer and in the electrolyte. In a first approximation, the gaseous oxygen concentration in the catalyst layer and the amount of FeN_*x*_C_*y*_ sties are assumed to be constant. The local ORR rate is equal to *k*_r_ × *C*_H_(*x*, *t*) where *k*_r_ is the ORR rate constant. The Fe dissolution rate from FeN_*x*_C_*y*_ sites is assumed to be the product of the ORR rate, *k*_r_ × *C*_H_(*x*, *t*), and a constant dissolution coefficient *k*_dis_ = 4.6 × 10^−5^.

In addition to the ORR, the reactions considered in the model are:

(a) The self-ionization of water, H_2_O ⇔ H^+^ + OH^−^ (*k*_fw_ = 1.4 × 10^−3^ M s^−1^, *k*_bw_ = 7 × 10^10^ M^−1^ s^−1^)

(b) The Fe precipitation: Fe^3+^ + 3H_2_O ⇔ Fe(OH)_3_ + 3H^+^ (solubility constant of Fe(OH)_3_, *K*_s_ = 1.0 × 10^−42^ at 70 °C, *k*_d_ = 0.01 s^−1^, *k*_p_ = 0.8 cm^6^ mol^−2^ s^−1^)

Thus, the set of partial differential equations describing the evolution of the concentration profiles in the catalyst layer can thus be expressed as:3
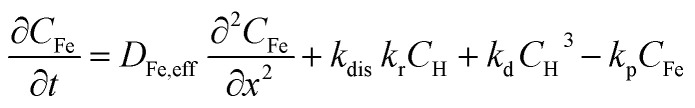
4
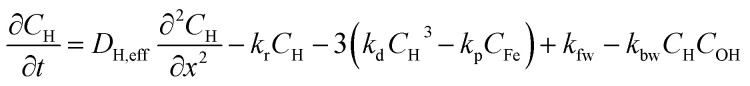
5
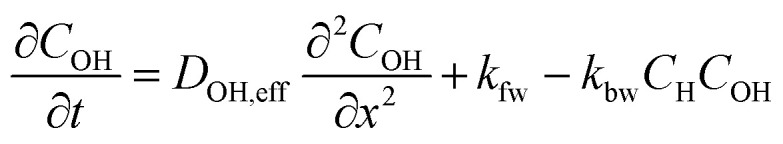
with *D*_Fe,eff_ = 6 × 10^−7^ cm^2^ s^−1^ and *D*_H,eff_ = 3.5 × 10^−5^ cm^2^ s^−1^.

The system of partial differential equations is solved for the initial conditions, at *t* = 0, *C*_Fe_ = 0 and *C*_H_ = 10^−1^ M, *C*_OH_ = 10^−13^ M. The following boundary conditions are used:

At the gas-phase/catalyst-layer interface at *x* = 0:
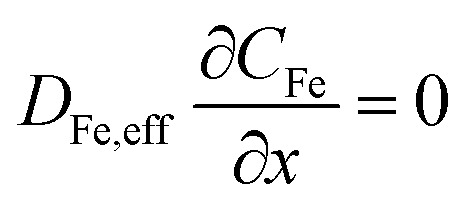

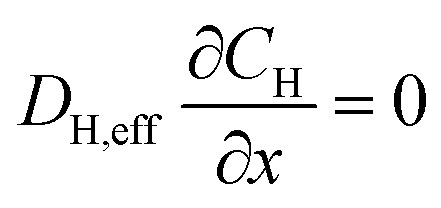

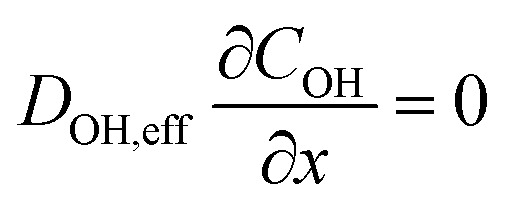


At the catalyst-layer/liquid-electrolyte interface at *x* = 40 μm:


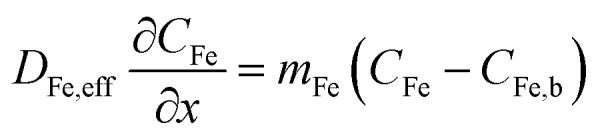
, where *C*_Fe,b_ = 10^−7^ M is the bulk concentration of dissolved iron in solution and *m*_Fe_ = 1.2 × 10^−3^ cm s^−1^ is the mass transfer coefficient of the Fe in the solution.


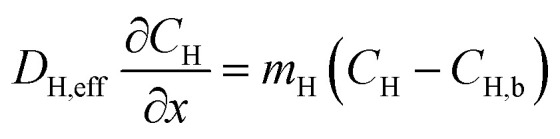
, where *C*_H,b_ = 10^−1^ M is the bulk concentration of proton in solution and *m*_H_ = 1.2 × 10^−2^ cm s^−1^ is the mass transfer coefficient of the protons.

The faradaic current is obtained by:

6 *j*_F_ = *F*∫*k*_r_*C*_H_(*x*,*t*)d*x*

where *F* = 96 485 C mol^−1^ is the Faraday constant.

## 3. Results

**Fig. 1:**
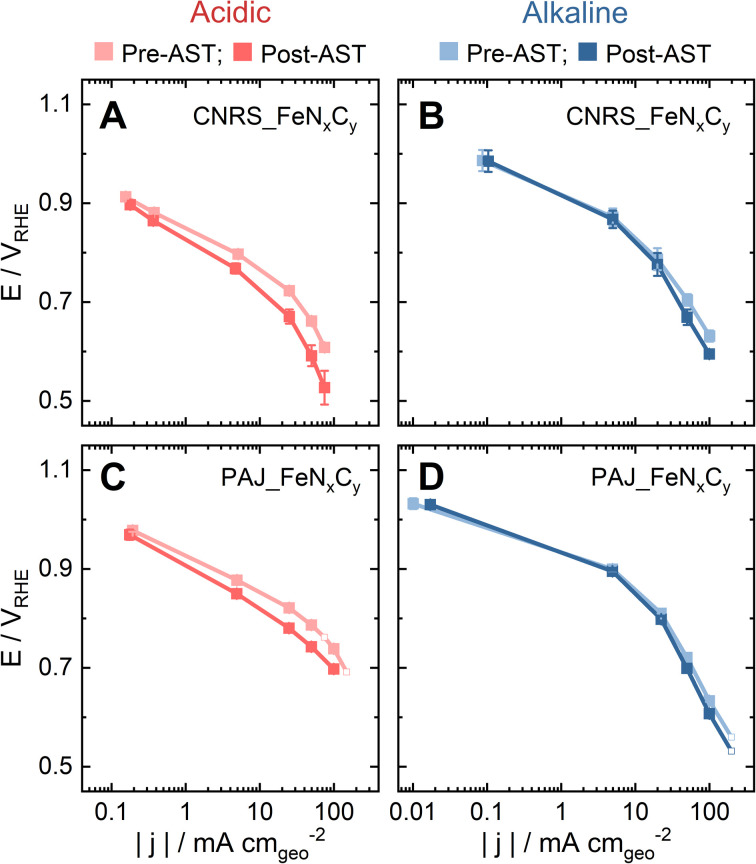
Tafel plots of the pristine (pre-AST) and degraded (post-AST) Fe–N–C gas diffusion electrodes in O_2_ at 70 ± 6 °C in the gas diffusion electrode half-cell (A & B) using CNRS_FeN_*x*_C_*y*_ as the catalyst (A) in 0.1 M HClO_4_ and (B) in 0.1 M NaOH; (C & D) using PAJ_FeN_*x*_C_*y*_ as the catalyst (C) in 0.1 M HClO_4_ and (D) in 0.1 M NaOH. The error bars represent the difference between the results of two experiments. The four blank square symbols show the data without repetition.

In order to investigate to which extent the *S*-number is an appropriate stability descriptor for Fe–N–C catalysts in relevant conditions in PEMFCs and AEMFCs, two benchmark FeN_*x*_C_*y*_-rich Fe–N–C catalysts (CNRS_FeN_*x*_C_*y*_ and PAJ_FeN_*x*_C_*y*_) were tested in O_2_-saturated 0.1 M HClO_4_ and 0.1 M NaOH at 70 ± 6 °C (Acidic-HT and Alkaline-HT, respectively) using a GDE-ICP-MS technique. The electrochemical protocols all involve an activity test pre-AST, a 200-cycle AST, and a repeated activity test post-AST. First, by comparing the pre-AST and post-AST Tafel plots, the drops in the ORR performance of the Fe–N–C catalyst layers can be observed in Fig. 1. Although the pristine ORR performance of both CNRS_FeN_*x*_C_*y*_ and PAJ_FeN_*x*_C_*y*_ varies with the electrolyte's pH value, the degradation in Acidic-HT (Fig. 1A and C) is clearly more severe than that in Alkaline-HT (Fig. 1B and D), agreeing with the theoretical and experimental results in the literature.^14,27^

**Fig. 2:**
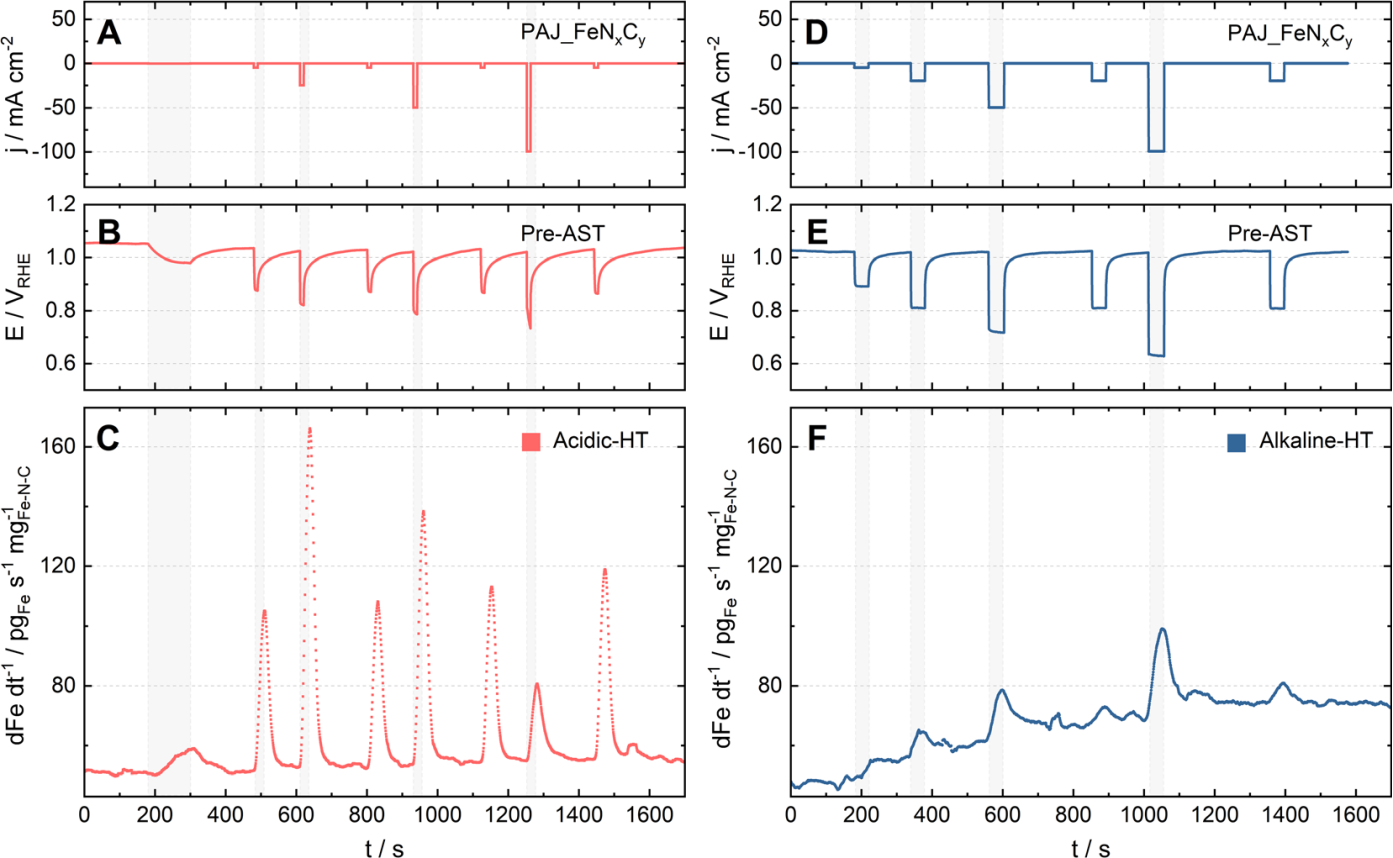
Online GDE-ICP-MS results during the pre-AST measurements for PAJ_FeN_*x*_C_*y*_ (A–C) in 0.1 M HClO_4_ at 70 ± 6 °C (Acidic-HT) and (D–F) in 0.1 M NaOH at HT (Alkaline-HT). (A & D) The current density profiles. (B & E) The potential profiles. (C & F) The corresponding online Fe dissolution profiles, which were normalized to the catalyst loading.

As one of the main degradation mechanisms, Fe demetallation from Fe–N–C catalysts has been shown using SFC-ICP-MS to be more dramatic in acidic than in alkaline media.^14^ Yet, the comparison in the literature was done at limited ORR current densities (|*j*| ≤ 10 mA cm^−2^), so this work uses GDE-ICP-MS to enable such a comparison at current densities up to −100 mA cm^−2^. For example, Fig. 2 presents the profiles of online Fe dissolution from PAJ_FeN_*x*_C_*y*_ for the Acidic-HT (see Fig. 2A–C) and Alkaline-HT (see Fig. 2D–F) conditions during the pre-AST activity tests, while the post-AST data is provided in Fig. S1 in the ESI.† As for CNRS_FeN_*x*_C_*y*_, the pre-AST and post-AST Fe dissolution profiles are shown in Fig. S2 and S3, respectively, in the ESI.† The dissolution peaks during the repeated chronopotentiometry (CP) steps at −5 mA cm^−2^ in Acidic-HT in between the CP steps at various current densities (marked with grey shades) barely vary (see Fig. 2C and S4, the latter of which is in the ESI†). This verifies that the trend of the dissolution peaks at different current densities is hardly influenced by the step order in the Acidic-HT protocol. Compared to the Acidic-HT condition, the signal-to-noise ratio was lower and the background increased faster in Alkaline-HT. The standard deviation of the Fe background signals at the first near OCP step (before the first CP step of interest) is, on average, 1.85 pg_Fe_ s^−1^ mg_Fe–N–C_^−1^ in Acidic-HT and 3.64 pg_Fe_ s^−1^ mg_Fe–N–C_^−1^ in Alkaline-HT (almost double of the value obtained in Acidic-HT). Hence, it is not straightforward to draw the same conclusion from the dissolution peaks during the repeated CP steps at −20 mA cm^−2^ in the pre-AST measurements in Alkaline-HT although they do not suggest otherwise. Nevertheless, except for the repeated CP steps at −20 mA cm^−2^, a step at a higher current density is always applied later to minimize the history effect. The trend of Fe dissolution in Acidic-HT is distinct from that in Alkaline-HT. In Alkaline-HT, the amount of the dissolved Fe species increased with the elevated current density. However, in Acidic-HT, the amount of the dissolved Fe species first climbed with the increased current density, reached a peak at −25 mA cm^−2^, and then decreased with the rising current density. Because the step durations in these two protocols had to be different, which has been explained in the 2.3 section, it is not straightforward to directly compare the Fe dissolution profiles (*e.g.*Fig. 2C and F). For easier comparison, the dissolution data will be henceforth shown as the *S*-numbers (see eqn (2)), which are calculated by dividing the number of electrons exchanged in the ORR by the number of the dissolved Fe species integrated from the dissolution profiles.

**Fig. 3:**
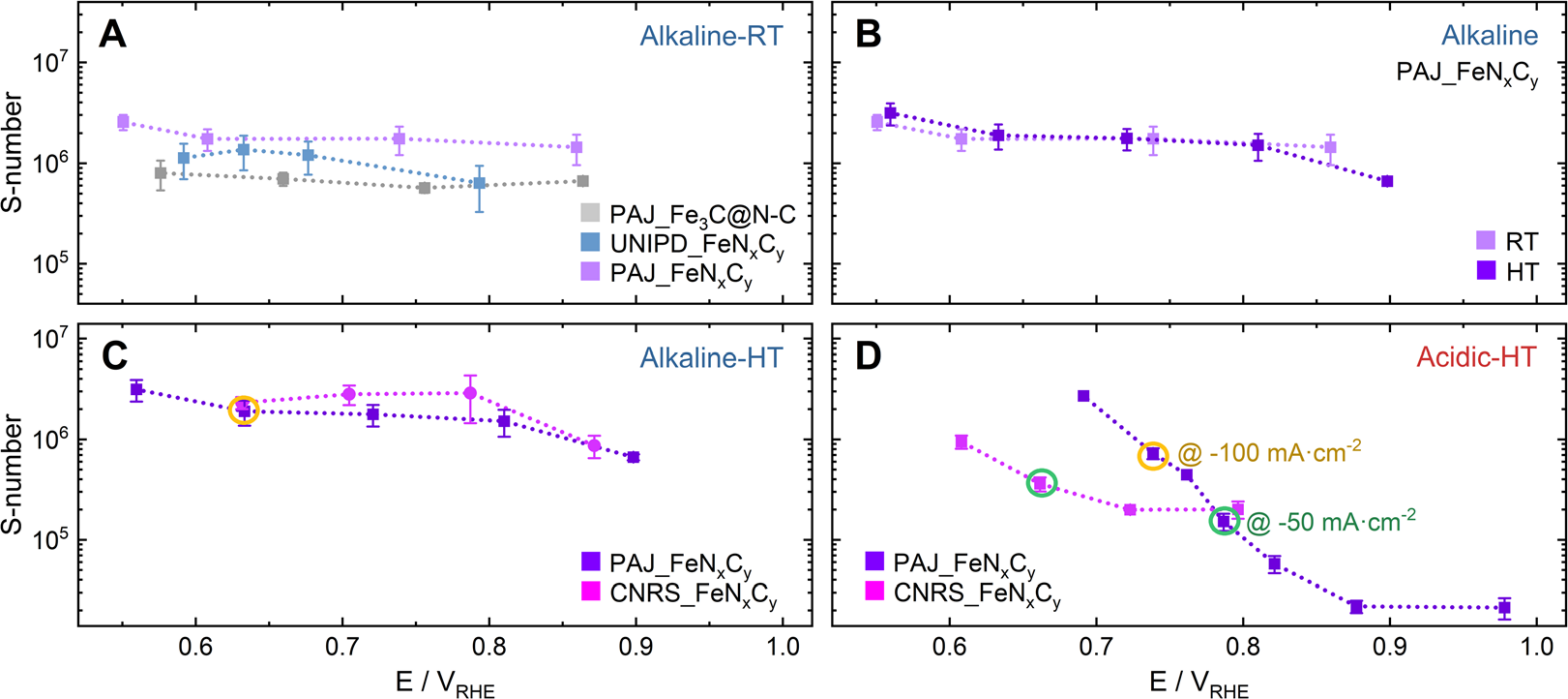
The plots of *S*-number against the electrochemical potential (*E* V_RHE_), where the *S*-number is defined as the number of electrons exchanged in the ORR per dissolved Fe cation. (A) A comparison of the *S*-numbers among three Fe–N–C catalysts, PAJ_Fe_3_C@N–C (light grey), UNIPD_FeN_*x*_C_*y*_ (light blue),^29^ and PAJ_FeN_*x*_C_*y*_ (light violet),^8^ in 0.1 M NaOH at room temperature (RT); (B) a comparison of the *S*-numbers of PAJ_FeN_*x*_C_*y*_ in 0.1 M NaOH at RT (light violet) and at 70 ± 6 °C (HT) (violet); (C & D) comparisons of the *S*-numbers between two Fe–N–C catalysts, CNRS_FeN_*x*_C_*y*_ (magenta) and PAJ_FeN_*x*_C_*y*_ (violet), (C) in 0.1 M NaOH at HT and (D) in 0.1 M HClO_4_ at HT. The green and orange circles (the latter: only for PAJ_FeN_*x*_C_*y*_) mark the data points corresponding to the ORR current density at −50 mA cm^−2^ and at −100 mA cm^−2^, respectively. The error bars represent the difference between the results of two experiments, and are mainly contributed by the unavoidable differences in the quality of the catalyst layers and the applied electrochemistry, as well as the detection limits of the ICP-MS. In (D), the two data points of PAJ_FeN_*x*_C_*y*_ without error bars (at 0.69 and 0.76 V_RHE_) are from only one measurement, but they also follow the trend of other data with error bars.

**Fig. 4:**
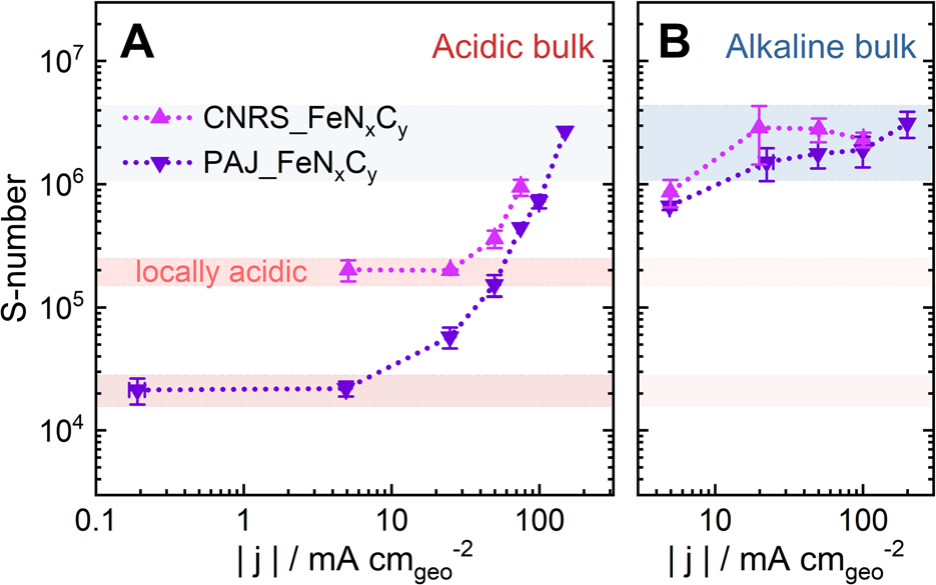
The plots of the *S*-numbers of two Fe–N–C catalysts, PAJ_FeN_*x*_C_*y*_ (violet) and CNRS_FeN_*x*_C_*y*_ (magenta), against the absolute value of the ORR current density |*j*| at 70 ± 6 °C (A) in 0.1 M HClO_4_ and (B) in 0.1 M NaOH. Note that the *S*-number data in (A and B) is the same set as in Fig. 3D and C, respectively. The error bars represent the differences between the results of two measurements. The red and blue shades suggest the ranges of the *S*-number of the Fe–N–C catalysts when the catalyst layers are dominantly acidic and alkaline, respectively.

The *S*-numbers in Fig. 3 and 4 were all calculated from the Fe dissolution data set during the pre-AST activity tests to ensure the *S*-numbers correspond to the Fe–N–C states that were still close to the pristine state, where the dominant Fe species had been identified before potential formations of other Fe species in a longer testing.^16,19,31^

A similar trend of the *S*-number is observed for two FeN_*x*_C_*y*_-rich Fe–N–C catalysts (PAJ_FeN_*x*_C_*y*_ (ref. 8) and UNIPD_FeN_*x*_C_*y*_ (ref. 29)) in Alkaline-RT (see Fig. 3A, light purple and light blue, respectively). Except for a slightly higher value for PAJ_FeN_*x*_C_*y*_ at 0.55 V_RHE_ and a slightly lower value for UNIPD_FeN_*x*_C_*y*_ at 0.79 V_RHE_, their *S*-numbers barely depend on the potential. Such a trend aligns with what was observed for an Fe–N–C catalyst rich with Fe_3_C@N–C (PAJ_Fe_3_C@N–C) in Alkaline-RT in our previous work (Fig. 3A, grey).^15^ The data of PAJ_FeN_*x*_C_*y*_ was published in another previous work in the form of electric charge-normalized Fe dissolution,^8^ and the *S*-number of UNIPD_FeN_*x*_C_*y*_ is newly reported here. The *S*-numbers of PAJ_Fe_3_C@N–C (0.58 < *E* < 0.86 V_RHE_), UNIPD_FeN_*x*_C_*y*_ (0.59 < *E* < 0.68 V_RHE_), and PAJ_FeN_*x*_C_*y*_ (0.61 < *E* < 0.86 V_RHE_), in Alkaline-RT are (0.8 ± 0.3) × 10^6^, (1.3 ± 0.6) × 10^6^, and (1.6 ± 0.7) ×10^6^, respectively. These reported *S*-numbers are of the same order of magnitude around 10^6^, and their differences are within the error bar. The *S*-number being 10^6^ means that on average every 10^6^ times the ORR charge transfer occurs, an Fe ion is dissolved from the catalyst layer.^15^ Additionally, the almost constant *S*-number interlinks the destabilization of the Fe species with the ORR catalytic cycle. Namely, the intermediates of the Fe species at one or more step(s) of the catalytic cycle are less stable than others, leading to a certain probability of Fe dissolution during each cycle. This degradation mechanism seems dominant for Fe–N–C catalysts in Alkaline-RT, as suggested by the unwavering trend of the *S*-number. However, the *S*-number's trend might vary with the conditions under which Fe–N–C catalysts are exposed.

Starting with the temperature effect on the trend of the *S*-number of Fe–N–C catalysts in alkaline media, Fig. 3B shows the *S*-numbers of PAJ_FeN_*x*_C_*y*_ in Alkaline-HT and Alkaline-RT (violet and light violet, respectively). Except for the data point at 0.9 V_RHE_ at HT, the rising temperature hardly changes the trend and value of the *S*-number or the ORR charge-normalized Fe dissolution, although the Fe concentration background before any electrochemical technique was indeed higher at HT than RT, suggesting a lower sensitivity detecting Fe dissolution at HT in base. Also, the post-AST dissolution data in Alkaline-HT has a high background, worsened signal-to-noise ratio, and greatly varied Fe dissolution peaks of the repeated steps at −20 mA cm^−2^ (see Fig. S1F and S3F in the ESI†), and thus such a data set is not suitable for calculating the *S*-number. In addition to PAJ_FeN_*x*_C_*y*_, another FeN_*x*_C_*y*_-rich Fe–N–C catalyst (CNRS_FeN_*x*_C_*y*_) was also tested in Alkaline-HT. The *S*-numbers of these two FeN_*x*_C_*y*_-rich Fe–N–C catalysts in Alkaline-HT follow the same trend (see Fig. 3C). The values stay almost constant in the *E* range between 0.81 and 0.63 V_RHE_, while at 0.885 ± 0.015 V_RHE_, they are two to three times lower than those between 0.81 and 0.63 V_RHE_. Therefore, the finding in Alkaline-RT that the amount of dissolved Fe during the ORR is highly correlated with the number of electrons exchanged in the ORR is still valid for FeN_*x*_C_*y*_-rich Fe–N–C catalysts at HT between 0.81 and 0.63 V_RHE_.

The influence of the electrolyte pH value on the stability of FeN_*x*_C_*y*_ sites at current densities from low values and up to −100 mA cm^−2^ is revealed by comparing the *S*-numbers of the FeN_*x*_C_*y*_-rich Fe–N–C catalysts in Alkaline-HT and Acidic-HT (compare Fig. 3C and D). Different from the trend of *S*-number observed in Alkaline-HT (see Fig. 3C), Fig. 3D reveals a trend of increasing stability (higher *S*-number) with decreasing potential (increasing current density). The *S*-numbers of CNRS_FeN_*x*_C_*y*_ in Acidic-HT (Fig. 3D, magenta) in the potential range 0.66 ≤ *E* ≤ 0.80 V_RHE_ are lower than its *S*-numbers in Alkaline-HT (Fig. 3C, magenta). This trend is also observed comparing the *S*-numbers of PAJ_FeN_*x*_C_*y*_ in Acidic-HT in the potential range 0.79 ≤ *E* ≤ 0.98 V_RHE_ and those in Alkaline-HT (Fig. 3D and C, violet). It is worth noting that the lower limits of the above-mentioned potential ranges (defined so that all *S*-numbers observed in acidic conditions are lower than *S*-numbers in alkaline conditions) are the potential values correspond to the ORR current density of −50 mA cm^−2^, marked with green circles in Fig. 3D. Because a lower *S*-number suggests worse stability, the results indicate that the FeN_*x*_C_*y*_ sites are much less stable in Acidic-HT at |*j*| ≤ 50 mA cm^−2^ than in Alkaline-HT. Moreover, when |*j*| is above 50 mA cm^−2^, such as 100 mA cm^−2^ for PAJ_FeN_*x*_C_*y*_ marked in orange circles in Fig. 3C and D, the *S*-number obtained in Acidic-HT is almost comparable to (only slightly lower than) that in Alkaline-HT. Hence, the FeN_*x*_C_*y*_ sites are generally less stable in Acidic-HT than in Alkaline-HT at the ORR current densities up to −100 mA cm^−2^, which at least partially explains the more severe degradation in the ORR performance after the AST in Acidic-HT *vs.* that after the AST in Alkaline-HT (Fig. 1).

Different from the steady *S*-numbers in Alkaline-RT and Alkaline-HT, the *S*-number obtained in Acidic-HT varies two orders of magnitude for PAJ_FeN_*x*_C_*y*_ in the *E* range 0.9–0.7 V_RHE_ and one order of magnitude for CNRS_FeN_*x*_C_*y*_ in the *E* range 0.8–0.6 V_RHE_ (see Fig. 3D). Such considerable changes in the *S*-number in Acidic-HT indicate a poor correlation between the rates of Fe dissolution and the ORR charge transfer, and/or underlying mechanisms that depend on the electrochemical potential and/or the ORR current density. As mentioned in the introduction, three potential mechanisms include the ROS attack, the potential-dependence or -independence of the oxidation state of FeN_4_C_*y*_ sites, and the increased local pH values at elevated ORR current densities. Because the impacts of the former two are specific to the species of FeN_*x*_C_*y*_ sites and the influence of the latter is more general for various FeN_*x*_C_*y*_ sites, in the following discussion section, we further explore how an elevated ORR current density may induce higher local pH values in the Fe–N–C catalyst layer in Acidic-HT and, subsequently, an increased *S*-number. For such a discussion, the *S*-number data in Fig. 3C and D is plotted against the absolute value of the ORR current density |*j*| in a log–log scale as the plots in Fig. 4B and A, respectively.

## 4. Discussion

The trend of the *S*-number of the Fe–N–C catalysts in Acidic-HT may be at least partially attributed to an increased local pH value during the ORR, which consumes H^+^. Our previous work^26^ reported that from pH = 1 at the catalyst-layer/liquid-electrolyte interface of a 60 μm Fe–N–C/Nafion catalyst layer in a GDE half-cell, the local pH value may rise to above pH = 8 at the gas/catalyst-layer interface at −15 mA cm^−2^ (ORR current density) at RT. On the other hand, at almost 0 mA cm^−2^ (0.75 V_RHE_) in Ar at RT, the calculated pH value in the catalyst layer barely shifts from pH = 1.^26^ This comparison of the local pH values at 0 and −15 mA cm^−2^ emphasizes the influence of the ORR current density on the local pH value in a catalyst layer contacting an acidic electrolyte. In Fig. 4A, in low-current-density regions, where the local environment of the active sites stays acidic, the *S*-numbers of the two FeN_*x*_C_*y*_-rich Fe–N–C catalysts differ by one order of magnitude, (2.2 ± 0.5) × 10^4^ for PAJ_FeN_*x*_C_*y*_ (0.2 ≤ |*j*| ≤ 5 mA cm^−2^) and (2.0 ± 0.4) × 10^5^ for CNRS_FeN_*x*_C_*y*_ (5 ≤ |*j*| ≤ 25 mA cm^−2^). However, as the current density rises, they follow the same trend where the values stay almost constant in the low-current-density regions, start rising at around −25 mA cm^−2^ for PAJ_FeN_*x*_C_*y*_ and at around −50 mA cm^−2^ for CNRS_FeN_*x*_C_*y*_, and then reach the order of magnitude observed in alkaline media around 10^6^ (marked as the blue shade in Fig. 4). This trend in Fig. 4A coincides with the hypothesis that a higher current density may result in a higher local pH value in the catalyst layer. Then, the increased local pH could lead to a higher stability of the HO-FeN_*x*_C_*y*_ species, an increased tendency for the dissolved Fe cations to redeposite as Fe oxide/hydroxide,^26^ and thus, a higher *S*-number, which eventually approaches that observed in alkaline media.

In contrast, the *S*-numbers of the Fe–N–C catalysts in alkaline media hardly vary with electrochemical potential (0.63 < *E* < 0.81 V_RHE_) or current density (20 < |*j*| < 100 mA cm^−2^) (see Fig. 4B). Although the local pH value may also increase in alkaline catalyst layers during the ORR, which produces OH^−^, the boundary condition at the catalyst-layer/electrolyte interface is already pH = 13. Increasing the pH value from pH = 13 to 14 requires a growth in the [OH^−^] from 0.1 to 1 M, calling for a higher ORR current density than a case where the local pH increases from pH = 1 to 2, corresponding to a drop in [H^+^] from 0.1 to 0.01 M. Thus, the impact of the ORR current density on the increase of the local pH value is less pronounced in alkaline media than in acidic media. Additionally, according to DFT calculations, the thermodynamically stable species at 0.6 V shifts from Fe^2+^ (Fe in FeN_*x*_C_*y*_ being less stable than free Fe^2+^) to HO–FeN_*x*_C_*y*_ when the pH value increases from only 1 to 2. In contrast, at pH = 13, the HO–FeN_*x*_C_*y*_ is already the dominant stable species.^27^ Consequently, the stability of the HO–FeN_*x*_C_*y*_ species can be greatly influenced by the ORR current density in acidic media but barely in alkaline media, agreeing with the trends of the *S*-number shown in Fig. 4.

**Fig. 5:**
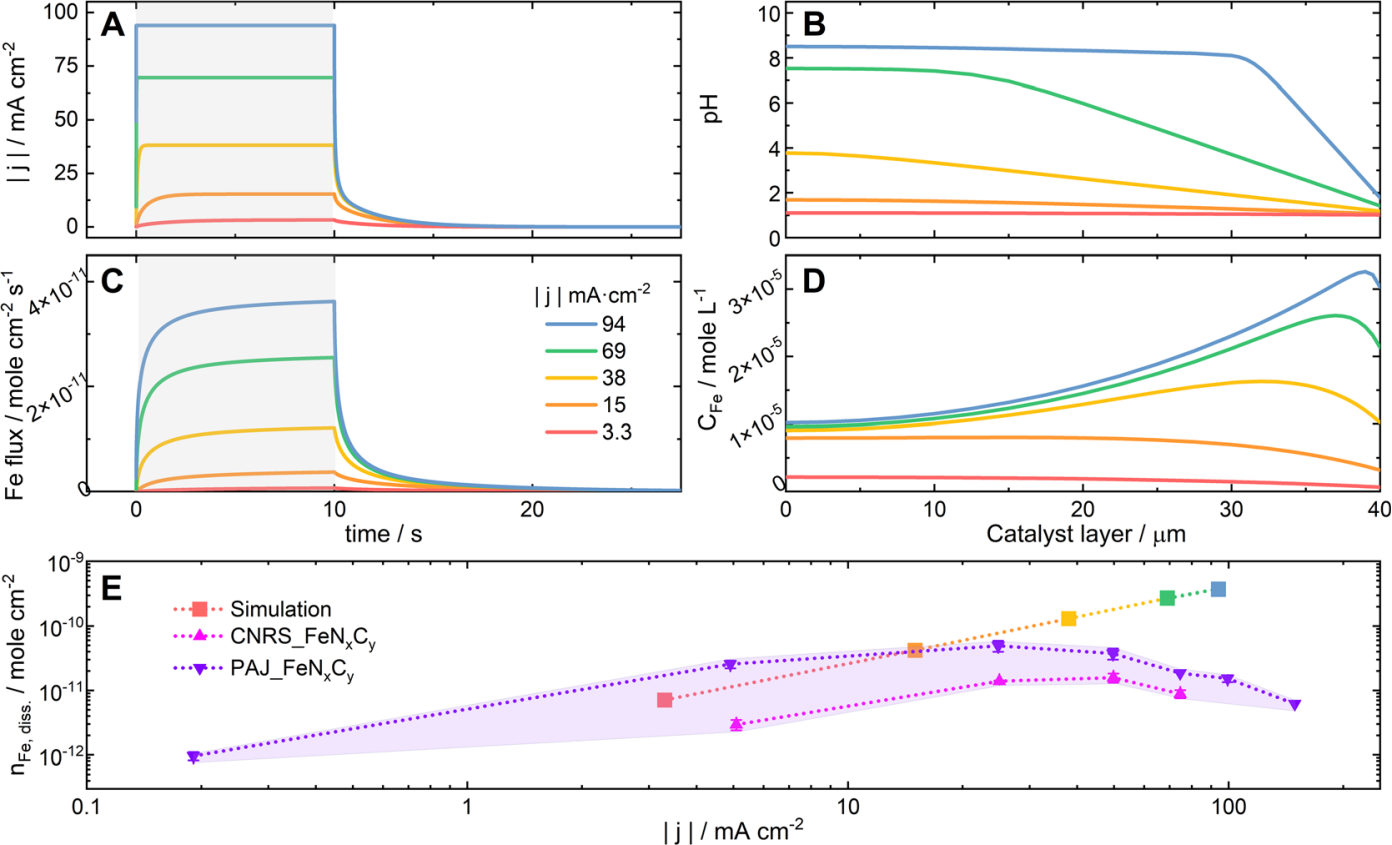
Simulation of a 40 μm Fe–N–C catalyst layer, one side of which is in contact with an electrolyte of pH = 1 and the other with a gas diffusion layer. (A) The current density profiles experienced by the catalyst layer, including 10-s chronopotentiometry (CP) steps at various current densities followed by OCP steps. (B) The resulting pH profiles in the catalyst layer at the end of the 10-s steps. (C) The Fe flux escaping from the catalyst layer during and after the 10-s steps. (D) The profiles of the concentration of Fe^*m*+^ (*C*_Fe_) in the catalyst layer at the end of the 10-s steps. (E) The amounts of the dissolved Fe during the 10-s steps at various current densities, which are integrated from the simulation data in (C) (square) and from the experimental data of CNRS_FeN_*x*_C_*y*_ (magenta, up triangle) and PAJ_FeN_*x*_C_*y*_ (violet, down triangle).

The two bases of the proposed hypothesis explaining the significant influence of the ORR current density on the stability of FeN_*x*_C_*y*_ active sites in Acidic-HT (or the trends of the *S*-numbers of FeN_*x*_C_*y*_-rich Fe–N–C catalysts in Acidic-HT in Fig. 4A) are that the elevated current density increases the local pH, and that the increased local pH stabilizes the FeN_*x*_C_*y*_ active sites and increases the tendency towards Fe oxide/hydroxide redeposition.^26^ While the latter is a well accepted knowledge of Fe–N–C catalysts,^14^ the former is a recently introduced concept,^26^ for which further validation is preferable before implementation. Hence, pH profiles of a 40-μm Fe–N–C catalyst layer are simulated at current densities up to −94 mA cm^−2^ with the initial pH value being 1. The kinetic model considered a 10-s CP step followed by an OCP step and was run independently 5 times with various current densities of the CP step (−3.3, −15, −38, −69, and −94 mA cm^−2^) (see Fig. 5A). The model takes the following reactions into account: the ORR, the self-ionization of water, the Fe precipitation, and the Fe dissolution from FeN_*x*_C_*y*_ sites (see Section 2.4). Additionally, the rate of Fe dissolution from Fe–N–C sites was set as the rate of ORR (*k*_r_ × *C*_H_) times a dissolution coefficient *k*_dis_ (see eqn (3) in Section 2.4) which is inversely proportional to *S*-number. Although *S*-number (or *k*_dis_) is supposed to be a function of pH, this is still a missing data set and thus cannot yet be input in the current kinetic model. At the current stage, for this simulation set, the value of *k*_dis_ is set as 4.6 × 10^−5^, the reciprocal of the *S*-number of PAJ_FeN_*x*_C_*y*_ at current densities below −5 mA cm^−2^ (2.2 × 10^4^, see Fig. 4A), to first show the increased local pH in the catalyst layer at elevated current densities. As a result, the simulated pH profiles in the catalyst layer at the end of the 10-s steps are shown in Fig. 5B, where the boundary conditions *x* = 0 and 40 μm correspond to the gas-phase/catalyst-layer interface and the catalyst-layer/liquid-electrolyte interface, respectively. The pH values in the catalyst layer generally rise with the increasing current density, verifying the hypothesis.

Although Fe precipitation is considered in the model, the pH-dependence of the *S*-number is not yet. The comparison between the simulation result and experimental data can reveal how the lack of such consideration leads to the discrepancy of the results. The Fe flux escaping from the catalyst layer is presented in Fig. 5C. Based on the model, the Fe flux expectedly increases with the elevating current density. By integrating the Fe flux with respect to time, the simulated amounts of the dissolved Fe during these five steps are obtained and plotted against the current density of the corresponding step in the log–log scale in Fig. 5E (square symbol, the color corresponding to that of the same set of simulation data in Fig. 5A–D). The simulated amounts of the dissolved Fe species during the 10-s step at −94 mA cm^−2^ is 3.7 × 10^−10^ mole_Fe_ cm^−2^ or 2.1 × 10^−2^ μg_Fe_ cm^−2^, which is around 0.3% of the Fe in an 1 cm^2^ catalyst layer loaded with 1 mg Fe–N–C catalyst that contains 0.65 wt% Fe. Because the amount of the dissolved Fe during the considered protocol is relatively minute, the model considers the FeN_*x*_C_*y*_ site density to be constant. From eqn (2), which was introduced in Section 2.3, the correlation between log|*j*| and log(*n*_Fe,diss._) can be written as eqn (7), suggesting that the theoretical slope of the simulation data in Fig. 5E is 1. Yet, the resulting slope of the simulation data is around 1.2. Its deviation from 1 can be at least partially attributed to the different mass transport (rate and direction) of the dissolved Fe species at varied current densities, which results from the different Fe concentration profiles in the catalyst layer (see Fig. 5D) and is considered in the kinetic model but not in eqn (7). For comparison, the experimentally acquired amounts of the dissolved Fe from PAJ_FeN_*x*_C_*y*_ and CNRS_FeN_*x*_C_*y*_ during the 10-s CP steps, which were used to calculate the *S*-numbers in Fig. 3D and 4A, are also plotted in Fig. 5E (violet and magenta triangular symbols, respectively).

7 log *n*_Fe,diss._ = log|*j*| + log(*t*/*e*/*N*_A_) − log(*S*-number)

Because the dependence of the *S*-number on the local pH value is not yet considered, the current model is limited to situations where the shift of the local pH values from 1 is still moderate. Otherwise, the simulation results would deviate from the experimental results. Indeed, when the current density is −3.3 mA cm^−2^, the pH values in the catalyst layer barely shift from 1 (Fig. 5B, red), and the simulated amount of the dissolved Fe (Fig. 5E, red) falls in between the experimental results of PAJ_FeN_*x*_C_*y*_ and CNRS_FeN_*x*_C_*y*_ (shaded in light violet for visualization). Moreover, in the low-current-density regions (|*j*| ≤ 5 mA cm^−2^ for PAJ_FeN_*x*_C_*y*_; |*j*| ≤ 25 mA cm^−2^ for CNRS_FeN_*x*_C_*y*_), the slopes of the experimental data curves in Fig. 5E are similar to that of the simulation data. Hence, in this current density region, the model of using a constant *S*-number is valid, agreeing with the observation in Fig. 4A that the *S*-numbers of the Fe–N–C catalysts stay almost constant at low current densities. Next, when the current density is −15 mA cm^−2^, the pH values in the catalyst layer have evidently shifted from 1 but are generally below 2 (Fig. 5B, orange). The slope of the experimental data curve of CNRS_FeN_*x*_C_*y*_ in Fig. 5E at −15 mA cm^−2^ stays similar to the simulation one, while that of PAJ_FeN_*x*_C_*y*_ has shifted lower. Interestingly, in Fig. 4A at −15 mA cm^−2^, the *S*-number of CNRS_FeN_*x*_C_*y*_ is still in its constant *S*-number region, while the *S*-number of PAJ_FeN_*x*_C_*y*_ has potentially shifted away from its constant *S*-number region. Namely, the model is not always (or not entirely) valid at such a current density. Furthermore, when the current density is above (not including) −15 mA cm^−2^, the pH values in the catalyst layer have largely shifted away from 1 (Fig. 5B, yellow, green, and blue). In these cases, the simulation results generally deviate from the experimental results in Fig. 5E, and the *S*-numbers of both catalysts deviate from the constant *S*-number regions observed at low current densities in Fig. 4A. Although this model cannot yet predict the amounts of dissolved Fe at high current densities in acidic media, it emphasizes the importance of considering the dependence of the *S*-number of FeN_*x*_C_*y*_ sites on the local pH value, and verifies that the pH values in the catalyst layer increase with the ORR current density (see Fig. 5B).

**Fig. 6:**
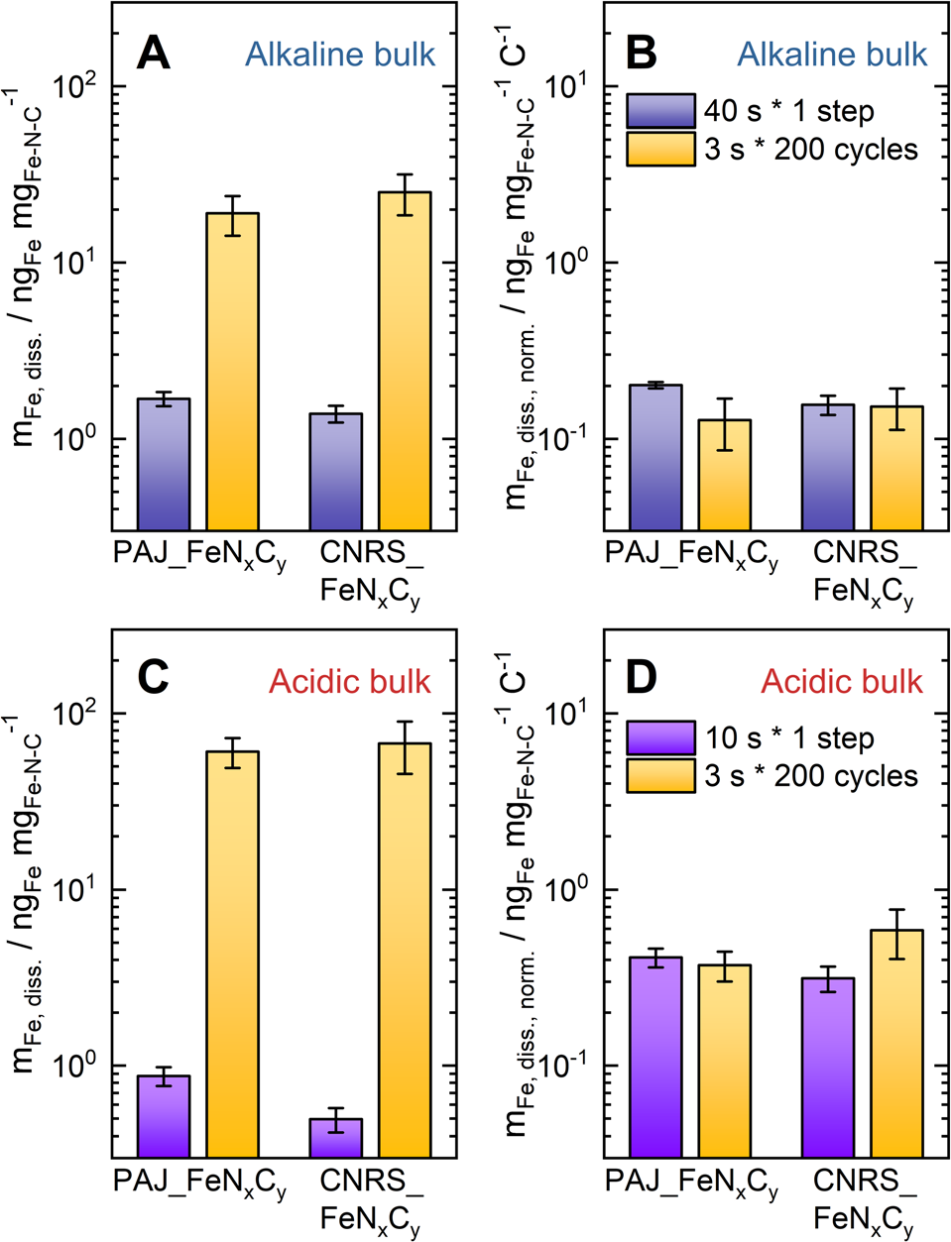
The Fe dissolution data of PAJ_FeN_*x*_C_*y*_ and CNRS_FeN_*x*_C_*y*_ during the 200-cycle AST (each cycle: 3 s at “*j*” mA cm^−2^ and 3 s at −0.1 mA cm^−2^, yellow bars) and the *t*-s galvanostatic step at “*j*” mA cm^−2^. The duration *t* of the galvanostatic step is 40 s in alkaline media (A & B, indigo bars) or 10 s in acidic media (C & D, violet bars), and the current density “*j*” is −75 mA cm^−2^ for CNRS_FeN_*x*_C_*y*_ in Acidic-HT, and −100 mA cm^−2^ for all the others (see Table 1). The amount of the dissolved Fe species has been normalized to the catalyst loading (A & C) or both the catalyst loading and the ORR charge (B & D). The error bars are equal to the differences between the results of the two measurements.

The linear correlation between the amount of the dissolved Fe species and the number of electrons exchanged in the ORR (the cumulative ORR faradaic charge divided by the elementary charge) at elevated current densities can still be examined by comparing the Fe dissolution during sub-protocols that applied the same current density, but implemented different step durations and cycle numbers. In the whole protocol, the 200-cycle AST and the step that applied the same elevated current density in the pre-AST activity test (−75 mA cm^−2^ for CNRS_FeN_*x*_C_*y*_ in Acidic-HT, and −100 mA cm^−2^ for all the others) are the most suitable for such a comparison. Hence, the Fe dissolution data of PAJ_FeN_*x*_C_*y*_ and CNRS_FeN_*x*_C_*y*_ in both alkaline and acidic media during these sub-protocols is summarized in Fig. 6.

The amounts of the dissolved Fe species normalized to the loading (*m*_Fe,diss._ ng_Fe_ mg_FeNC_^−1^) during these sub-protocols in alkaline and acidic media are presented in Fig. 6A and C, respectively. Fig. 6A shows that the values of *m*_Fe,diss._ in alkaline media during the AST (yellow) are around one order of magnitude higher than those during the 40-s step (indigo). However, after further normalizing the *m*_Fe,diss._ values to the ORR charges (see *m*_Fe,diss.,norm._ ng_Fe_ mg_FeNC_^−1^ C^−1^ in Fig. 6B), their values are almost the same for CNRS_FeN_*x*_C_*y*_ and very close for PAJ_FeN_*x*_C_*y*_, considering the error bars. Eqn (8) shows that *m*_Fe,diss.,norm._ is inversely proportional to the *S*-number. Namely, their *S*-numbers during the AST are comparable to those pre-AST. This analysis verifies again the correlation between the amount of the dissolved Fe species and the number of electrons exchanged in the ORR, which has been reported for PAJ_Fe_3_C@N–C and CNRS_FeN_*x*_C_*y*_ in alkaline media at RT.^15^ The slightly lower *m*_Fe,diss.,norm._ for PAJ_FeN_*x*_C_*y*_ during the AST than that during the 40-s step can be attributed to a minor history effect. As for the acidic condition, Fig. 6C shows that the *m*_Fe,diss._ values during the AST (yellow) become around two orders of magnitude larger than those during the corresponding 10-s step (violet). Once more normalizing the *m*_Fe,diss._ values to the ORR charges (see Fig. 6D), the resulting values are similar for PAJ_FeN_*x*_C_*y*_ and of the same magnitude for CNRS_FeN_*x*_C_*y*_. In other words, their *S*-numbers during the AST in Acidic-HT are similar to those obtained at the pre-AST galvanostatic step at the corresponding current density. This analysis suggests that in acidic media, the number of electrons exchanged in the ORR is also proportional to the amount of dissolved Fe from the FeN_*x*_C_*y*_-rich Fe–N–C catalysts when the applied current densities are the same. The equal ORR rates may lead to similar local pH values in the catalyst layers and, in turn, comparable stabilities of the FeN_*x*_C_*y*_ sites and tendencies of Fe oxide/hydroxide redeposition. Consequently, the *S*-number for Fe–N–C catalysts in acidic media can also be meaningful when the impact of the applied current density on the local pH in catalyst layers and thus the *S*-number is considered.8
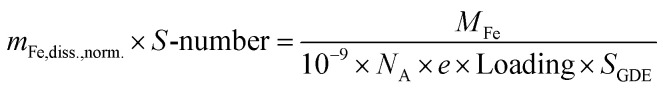
where *N*_A_, *e*, *M*_Fe_, and *S*_GDE_ refer to the Avogadro constant, the elementary charge, the molar mass of Fe, and the geometric area of the GDE sample, respectively.

## 5. Conclusions and outlook

This work verifies the remarkable correlation between the rates of Fe dissolution and ORR charge transfer in alkaline media at both RT and 70 °C, regardless of the dominant Fe species, the electrochemical potential (between 0.81 and 0.63 V_RHE_), and the current density (up to −100 mA cm^−2^) in the studied ranges. The resulting unwavering *S*-number in alkaline media suggests a certain probability of Fe leaching during each ORR catalytic cycle as a result of one or more intermediates of the Fe active sites present in both the ORR catalytic cycle and Fe dissolution process.^32^ On the other hand, in acidic media at 70 °C, the almost constant *S*-number is so far only observed in a low-current-density region or for protocols at the same elevated current density. We attribute the dependence of the *S*-number on the current density in acidic media at least partially to the rising pH value in the catalyst layer induced by elevated ORR current densities, evidenced by kinetic modeling.

On the basis of this work, future works may further develop the following topics.

(a) Consider the pH-dependence of the *S*-number in the kinetic model: first, the *S*-numbers of Fe–N–C catalysts in different pH media at low current densities (|*j*| ≤ 5 mA cm^−2^) may be obtained using SFC-ICP-MS, which is more time-efficient than GDE-ICP-MS. Next, the experimentally acquired *S*-number as a function of pH can be incorporated into the kinetic model developed in this work to predict the amount of dissolved Fe at an elevated current density in acidic electrolytes. Then, the simulation results can be compared with the Fe dissolution data obtained with GDE-ICP-MS to further discuss the degradation mechanisms of Fe–N–C catalysts at elevated current densities. Note that for a long-term operation, the change in the site density over time should be considered in the kinetic model.

(b) Experimentally probe the local pH in (or close to) GDE samples during the ORR: for example, the probe electrode in scanning electrochemical microscopy (SECM) can be developed as a voltammetric pH sensor to reveal the local pH in close proximity to a GDE sample during the ORR.^33^ Yet, SECM could not reveal the pH within the catalyst layers. On the other hand, using confocal microscopy and a pH-sensitive two-color fluorescent dye can offer the local pH profiles in catalyst layers, but it may be questionable if and how the dye interferes with the electrochemistry.^34^ Hence, the method development for probing the local pH “in” catalyst layers, especially the thick ones, at elevated current densities without interfering with the electrochemistry is still required. In such future works, the experimental results and simulation data can be compared and discussed.

(c) Investigate the trend of *S*-number during a long-term operation: because the composition of the Fe species may vary over a long-term operation, the stability of Fe–N–C catalysts may as well. While the pre- and post-AST *S*-numbers of Fe–N–C catalysts in Acidic-HT are compared and discussed in Fig. S5 and the Supporting discussion in the ESI,† the *S*-number of Fe–N–C catalysts mainly with Fe oxides or Fe hydroxides should be obtained in a following work before the evolution trend of *S*-number of benchmark Fe–N–C catalysts during a long-term operation may be meaningfully discussed.

(d) Compare Fe dissolution from Fe–N–C catalysts in O_2_- and Ar-purged acidic media in a GDE setup: such a comparison has been carried out in acidic media in a rotating disk electrode half-cell (RDE),^20^ and in alkaline media in both RDE and GDE half-cells.^15,19^ Yet, it has not been done in acidic media in a GDE setup, where elevated ORR current densities are reachable, while such a study may provide more fundamental insights to how Fe–N–C catalysts degrade.

(e) Use other representative single-atom catalysts^18,35–44^ to further benchmark and develop best practices of the application of the *S*-number to generalize the findings of this work.

## Data availability

All supporting data are provided in this paper.

## Author contributions

Yu-Ping Ku: GDE-ICP-MS experimental design and measurement, data curation, formal analysis, scientific discussion, writing of original draft Kavita Kumar: GDE-ICP-MS experimental design and measurement, scientific discussion, editing Pierre Antoine Bonnefont: kinetic modeling, scientific discussion, editing Li Jiao: synthesis of the Fe–N–C catalyst CNRS_FeN_*x*_C_*y*_; Marco Mazzucato: synthesis of the Fe–N–C catalyst UNIPD_FeN_*x*_C_*y*_ Christian Durante: scientific discussion, editing Frédéric Jaouen: scientific discussion, editing Serhiy Cherevko: project conceptualization and administration, validation, scientific discussion, editing.

## Conflicts of interest

The authors declare no conflict of interest.

## Supplementary Material

SC-OLF-D5SC00547G-s001
