# Supplementary material for: Establishing the stability number descriptor for Fe–N–C fuel cell electrocatalysts
Source: Chem Sci. 2025 Apr 14;16(20):8697–710. doi: 10.1039/d5sc00547g (PMC12016105; doi:10.1039/d5sc00547g)
Supplement: SC-016-D5SC00547G-s001 [file SC-016-D5SC00547G-s001.pdf]

Supplementary Information (SI) for Chemical Science.
This journal is © The Royal Society of Chemistry 2025

# - Supporting Information -

Establishing Stability Number Descriptor for Fe-N-C Fuel Cell Electrocatalysts

Yu-Ping Ku[a,b,*], Kavita Kumar[a],Antoine Bonnefont[c], Li Jiao[d], Marco Mazzucato[e], Christian Durante[e], Frédéric Jaouen[d], Serhiy Cherevko[a,*]

[a] Forschungszentrum Jülich GmbH, Helmholtz-Institute Erlangen-Nürnberg for Renewable Energy (IET-2), Cauerstraße 1, 91058 Erlangen, Germany

[b] Friedrich-Alexander University Erlangen-Nürnberg, Department of Chemical and Biological Engineering, Egerlandstraße 3, 91058 Erlangen, Germany

[c] Univ. Grenoble Alpes, Univ. Savoie-Mont-Blanc, CNRS, Grenoble-INP, LEPMI, 38000 Grenoble, France

[d] Institut Charles Gerhardt Montpellier, Univ. Montpellier, CNRS, ENSCM, 1919 route de Mende, F-34293 Montpellier, France

[e] University of Padova, Department of Chemical Sciences, via Marzolo 1, 35131, Padova, Italy

[*] corresponding authors: yupingku427@gmail.com, s.cherevko@fz-juelich.de

Table of contents

1. Supporting results – online Fe dissolution data 3

2. Supporting discussion 5

3. References 6

1. Supporting results – online Fe dissolution data

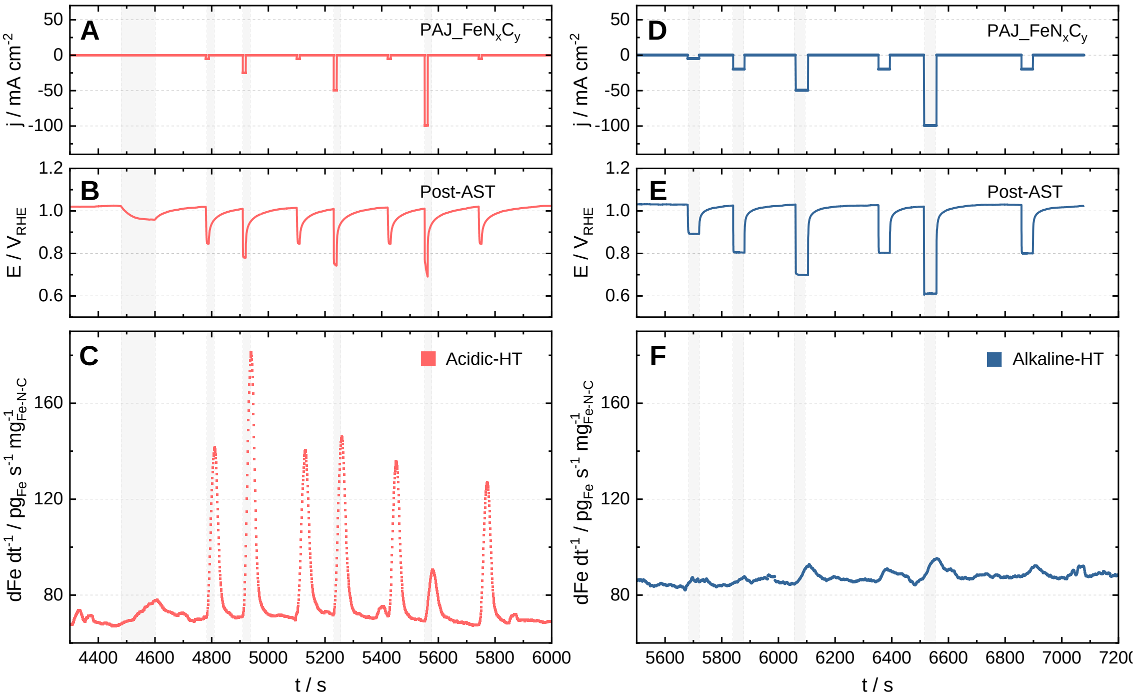

Figure S1. Online GDE-ICP-MS results during the post-AST measurements for PAJ_FeN$_x$C$_y$ (A, B, & C) in 0.1 M $HClO_4$ at 70 ± 6 °C (Acidic-HT) and (D, E, & F) in 0.1 M NaOH at HT (Alkaline-HT). (A & D) The current density profiles. (B & E) The potential profiles. (C & F) The corresponding online Fe dissolution profiles, which were normalized to the catalyst loading.

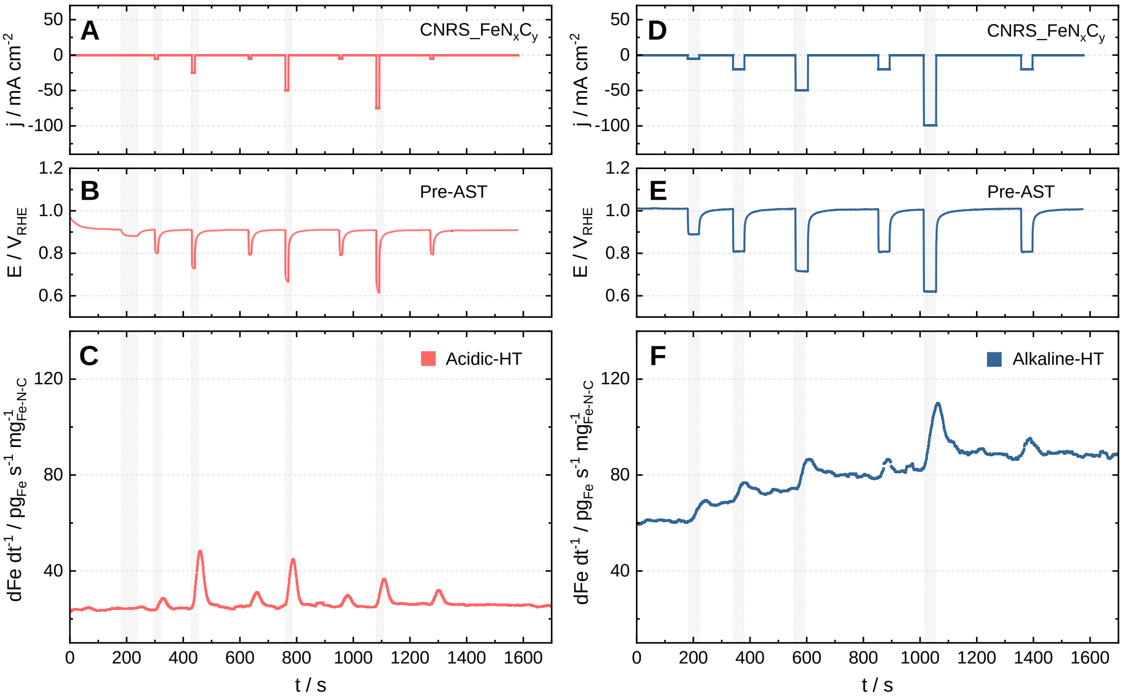

Figure S2. Online GDE-ICP-MS results during the pre-AST measurements for CNRS_FeN$_x$C$_y$ (A, B, & C) in 0.1 M $HClO_4$ at 70 ± 6 °C (Acidic-HT) and (D, E, & F) in 0.1 M NaOH at HT (Alkaline-HT). (A & D) The current density profiles. (B & E) The potential profiles. (C & F) The corresponding online Fe dissolution profiles, which were normalized to the catalyst loading.

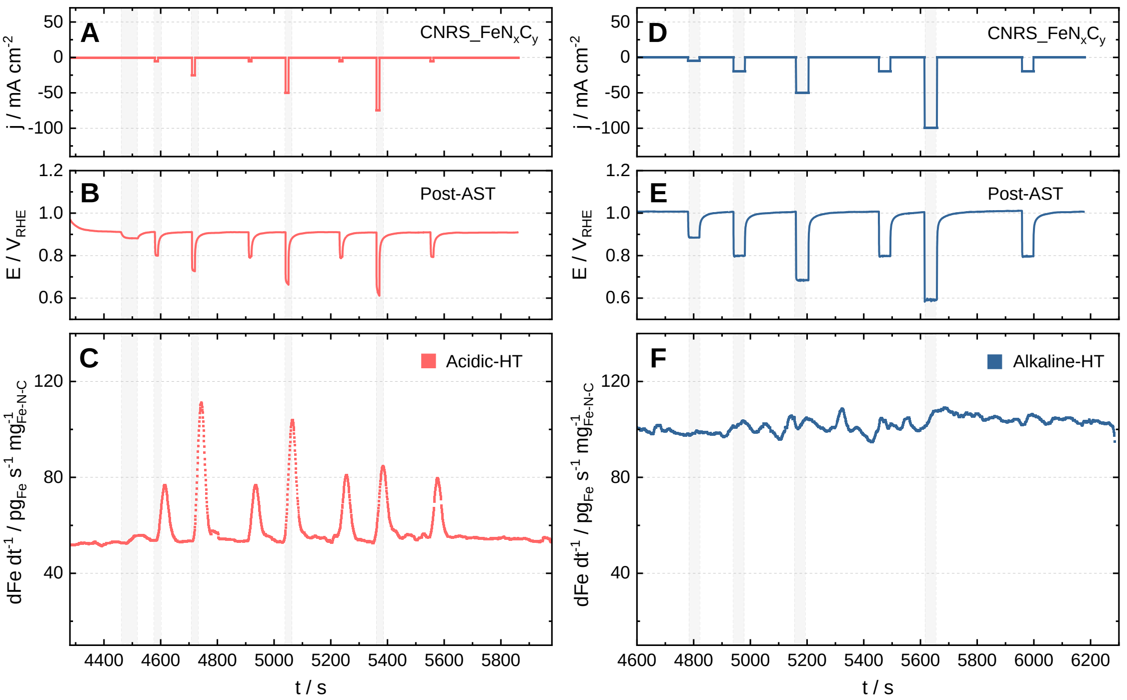

Figure S3. Online GDE-ICP-MS results during the post-AST measurements for CNRS_FeN$_x$C$_y$ (A, B, & C) in 0.1 M HClO$_4$ at 70 ± 6 °C (Acidic-HT) and (D, E, & F) in 0.1 M NaOH at HT (Alkaline-HT). (A & D) The current density profiles. (B & E) The potential profiles. (C & F) The corresponding online Fe dissolution profiles, which were normalized to the catalyst loading.

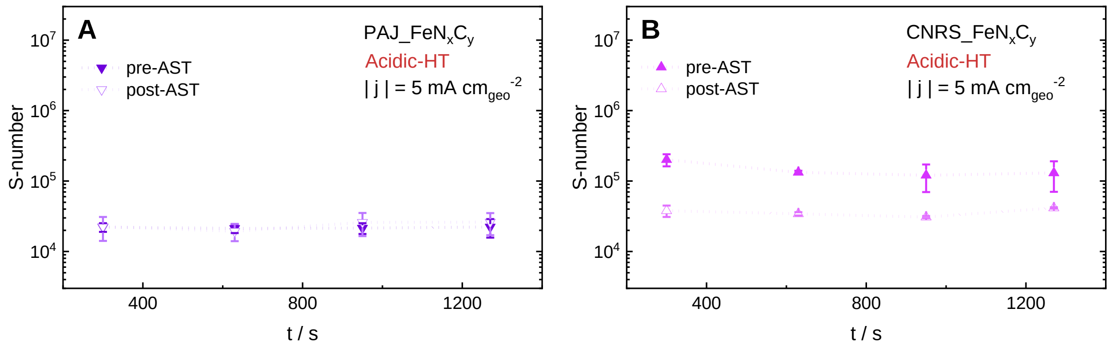

Figure S4. The S-number at the repeated CP steps at | *j* | = 5 mA·cm^{-2} in Acidic-HT for (A) PAJ_FeN$_x$C$_y$ and (B) CNRS_FeN$_x$C$_y$. The results from the pre-AST and post-AST measurements are presented with solid and open symbols, respectively.

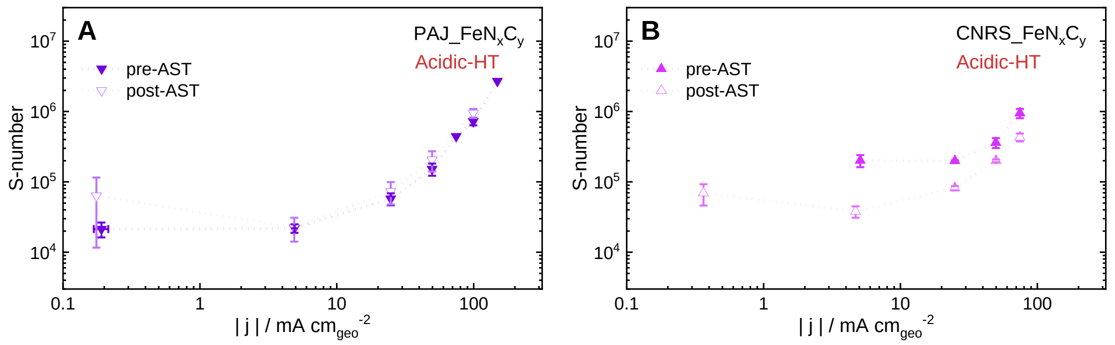

Figure S5. The S-number at various current densities in Acidic-HT for (A) PAJ_FeN_xC_y and (B) CNRS_FeN_xC_y. The results from the pre-AST and post-AST measurements are presented with solid and open symbols, respectively.

2. Supporting discussion

In Figure S5, the trends of the post-AST S-number (open symbols) in Acidic-HT are presented and compared with the pre-AST results (solid symbols). For PAJ_FeN_xC_y (see Figure S5A), the S-number barely shifted after the AST, except for the data at -0.18 mA·cm^{-2}, of which the error bar increased potentially due to the dropped detection sensitivity after the AST. As for CNRS_FeN_xC_y (see Figure S5B), the S-number generally dropped after the AST. The change in S-number over an AST or a long-term operation may be attributed to the change in the composition of the Fe species in CNRS_FeN_xC_y. In a previous work,[1] a similarly synthesized Fe-N-C catalyst (noted as $Fe_{0.5}$-950(10) in Ref.[1]) was applied in the cathode of a PEMFC that underwent a potential hold at 0.5 V for 50 hours. The slight difference between the synthesis protocols of CNRS_FeN_xC_y and $Fe_{0.5}$-950(10) was the duration of the second pyrolysis at 950℃ in NH_3, which was 5 and 10 minutes, respectively. Also, another previous work has shown that CNRS_FeN_xC_y (noted as $Fe_{0.5}$-950(5) in Ref.[2]) and $Fe_{0.5}$-950(10) perform comparably in PEMFCs, and have very similar fingerprints in their Mössbauer spectra and X-ray absorption spectra.[2] In the Extended Data Fig. 7 in Ref.[1], the Mössbauer spectra of $Fe_{0.5}$-950(10) cathode before and after the 50-hour potential hold show the decrease of sites S1 (FeN_4C_{12}) and the increase of ferric oxides during the operation. Such a change in the Fe species can

also be expected in CNRS_FeN_xC_y during the AST because the synthesis protocols, the Fe species, and the PEMFC performance of the two catalysts (CNRS_FeN_xC_y and $Fe_{0.5}$-950(10)) are all nearly the same. Hence, the dropped S-numbers after the AST suggest that the S-number of ferric oxides may be lower than that of sites S1, which may be verified by testing Fe-N-C catalysts mainly with ferric oxides, rather than highly mixed Fe species, in a following work.

3. References

1. Li, J.; Sougrati, M. T.; Zitolo, A.; Ablett, J. M.; Oğuz, I. C.; Mineva, T.; Matanovic, I.; Atanassov, P.; Huang, Y.; Zenyuk, I.; Di Cicco, A.; Kumar, K.; Dubau, L.; Maillard, F.; Dražić, G.; Jaouen, F. Identification of durable and non-durable FeN_x sites in Fe–N–C materials for proton exchange membrane fuel cells. *Nature Catalysis* **2021,** *4* (1), 10-19.

2. Zitolo, A.; Goellner, V.; Armel, V.; Sougrati, M.-T.; Mineva, T.; Stievano, L.; Fonda, E.; Jaouen, F. Identification of catalytic sites for oxygen reduction in iron- and nitrogen-doped graphene materials. *Nature Materials* **2015,** *14* (9), 937-942.
